# Postgenomics Characterization of an Essential Genetic Determinant of Mammary Pathogenic *Escherichia coli*

**DOI:** 10.1128/mBio.00423-18

**Published:** 2018-04-03

**Authors:** Shlomo E. Blum, Robert J. Goldstone, James P. R. Connolly, Maryline Répérant-Ferter, Pierre Germon, Neil F. Inglis, Oleg Krifucks, Shubham Mathur, Erin Manson, Kevin Mclean, Pascal Rainard, Andrew J. Roe, Gabriel Leitner, David G. E. Smith

**Affiliations:** a National Mastitis Center, Division of Bacteriology, Kimron Veterinary Institute, Bet Dagan, Israel; b IB3, Heriot Watt University, Edinburgh, United Kingdom; c Institute of Infection, Immunity & Inflammation, College of Medical, Veterinary and Life Sciences, University of Glasgow, Glasgow, United Kingdom; d ISP, INRA, Université Tours, Nouzilly, France; e Moredun Research Institute, Edinburgh, Midlothian, United Kingdom; GSK Vaccines

**Keywords:** *Escherichia coli*, bovine, ferric citrate, mammary gland, mastitis, milk, pathogenesis, whole-genome sequencing

## Abstract

Escherichia coli are major bacterial pathogens causing bovine mastitis, a disease of great economic impact on dairy production worldwide. This work aimed to study the virulence determinants of mammary pathogenic E. coli (MPEC). By whole-genome sequencing analysis of 40 MPEC and 22 environmental (“dairy-farm” E. coli [DFEC]) strains, we found that only the *fec* locus (*fecIRABCDE*) for ferric dicitrate uptake was present in the core genome of MPEC and that it was absent in DFEC genomes (*P* < 0.05). Expression of the FecA receptor in the outer membrane was shown to be citrate dependent by mass spectrometry. FecA was overexpressed when bacteria were grown in milk. Transcription of the *fecA* gene and of the inner membrane transport component *fecB* gene was upregulated in bacteria recovered from experimental intramammary infection. The presence of the *fec* system was shown to affect the ability of E. coli to grow in milk. While the rate of growth in milk of *fec*-positive (*fec*^+^) DFEC was similar to that of MPEC, it was significantly lower in DFEC lacking *fec*. Furthermore, deletion of *fec* reduced the rate of growth in milk of MPEC strain P4, whereas *fec*-transformed non-mammary gland-pathogenic DFEC strain K71 gained the phenotype of the level of growth in milk observed in MPEC. The role of *fec* in E. coli intramammary pathogenicity was investigated *in vivo* in cows, with results showing that an MPEC P4 mutant lacking *fec* lost its ability to induce mastitis, whereas the *fec*^+^ DFEC K71 mutant was able to trigger intramammary inflammation. For the first time, a single molecular locus was shown to be crucial in MPEC pathogenicity.

## IMPORTANCE

Bovine mastitis is the major infectious disease in dairy cows and the leading cause of economic loss to the global dairy industry, directly contributing to the price of dairy products on supermarket shelves and the financial hardships suffered by dairy farmers. Mastitis is also the leading reason for the use of antibiotics in dairy farms. Good farm management practices in many countries have dramatically reduced the incidence of contagious mastitis; however, the problems associated with the incidence of environmental mastitis caused by bacteria such as Escherichia coli have proven intractable. E. coli bacteria cause acute mastitis, which affects the health and welfare of cows and in extreme cases may be fatal. Here we show for the first time that the pathogenicity of E. coli causing mastitis in cows is highly dependent on the *fecIRABCDE* ferric citrate uptake system that allows the bacterium to capture iron from citrate. The Fec system is highly expressed during infection in the bovine udder and is ubiquitous in and necessary for the E. coli bacteria that cause mammary infections in cattle. These results have far-reaching implications, raising the possibility that mastitis may be controllable by targeting this system.

## INTRODUCTION

Bacteria in the species Escherichia coli are a principal causative agent of bovine mastitis (mammary pathogenic E. coli [MPEC]), costing dairy farmers in the European Union an estimated €2 billion per year, with a similar economic burden for the United States, where each case of mastitis has been estimated to cost $444 (1). The infection is usually—in upwards of 85% of cases—treated with antimicrobial therapy, which may contribute to the rise and spread of antibiotic-resistant bacteria (2) as well as preventing the use of milk from the affected cow in dairy products for several days following treatment. In addition to the requirement to withhold milk following antibiotic usage, there are also long-term alterations in milk quality following episodes of E. coli mastitis, regardless of the treatment regimen (3). Furthermore, and despite the use of antibiotics, mastitis carries a substantial risk of mortality or severe morbidity resulting in culling of the affected animal (4), a factor which adds to the cost of this disease with respect to the economic and psychological well-being of dairy farmers, who often form strong emotional attachments with their livestock (5). Clearly, any management practice or treatment which achieves one or more of the goals of (i) reducing the incidence of mastitis without contributing to antibiotic resistance, (ii) reducing the risk of morbidity or mortality following infection, and (iii) reducing the withholding period for milk to be used in dairy products following treatment will be of great benefit both to the dairy industry and to consumers.

Iron is a critical micronutrient for the growth of most life-forms, acting as a necessary cofactor for a variety of enzymes. In bovine milk, much of the iron is bound to various proteins such as lactoferrin and transferrin; however, milk also contains substantial concentrations of citrate, which also binds iron with high affinity (6). In fact, the molar concentration of citrate in milk (approximately 10 mM) greatly exceeds that of other iron chelating agents (7). To extract iron from milk, it makes logical sense that bacteria must possess ways to release iron from these chelating agents, and strains of E. coli are known to encode different complements of such iron acquisition systems (8). These systems are known to impact the fitness and ability of distinct E. coli strains to cause other diseases (9). Given the high concentrations of citrate in bovine milk, it is pertinent that some E. coli strains possess a specific system to capture iron from ferric citrate (Fe-cit). This system, encoded by the *fecIRABCDE* locus, binds and translocates Fe-cit at the outer membrane via FecA and internalizes Fe via FecB, FecC, FecD, and FecE. The binding of Fe-cit to FecA also transduces a signal, via the response transducer FecR, to the alternative sigma factor FecI, resulting in upregulation of the *fecABCDE* genes. FecA expression has been detected in E. coli strains isolated from mastitis before (10), and in a recent population level analysis, we identified the Fec system as a core MPEC determinant for the commonest lineage of E. coli implicated in mastitis (11). In this study, we experimentally explored the contribution of the Fec system to E. coli mastitis.

## RESULTS

### The Fec system defined MPEC at the genomic level in a farm-controlled study.

**FIG 1:**
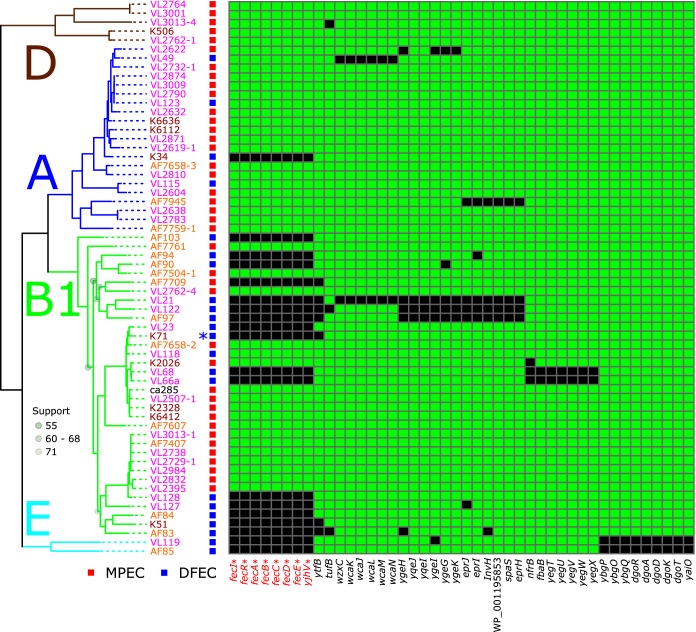
Presence/absence diagram showing the distribution of 45 genes identified as the specific core MPEC genome alongside the phylogenetic relationships between the strains. Phylogeny data are based on maximum likelihood inferences of nucleotide sequences for genes shared by all strains (1,000 bootstrap replicates, 2,838,669 alignment positions). Data representing bootstrap support for nodes below 90% are labeled. Phylogroup assignments are inferred from a larger tree comprising 4,022 E. coli genome sequences (shown in Fig. S1). Genes with statistically significant association with MPEC (corrected *P* < 0.05) are indicated with an asterisk. K71—used in this work—is highlighted with an asterisk next to its name.

In a recent publication considering MPEC at the population level, we implemented a novel method to detect genes in the MPEC population which we postulated are critical for their lifestyle. This method employed defining of genes that were present in the core genome of MPEC and yet absent in the core genome of closely related strains from the more widely available E. coli genomic resource. In this way, we detected just three genetic loci which were core in—and presumably necessary for—MPEC and yet were not always present in other E. coli strains and therefore were probably not required for the fitness of E. coli in general (11). Here, we employed a similar analysis; however, rather than considering close phylogenetic relatives of MPEC, we systematically collected 62 E. coli samples both from the milk of infected cows and from the dairy farm environment (dairy farm E. coli [DFEC]). These DFEC samples were collected from the same farm locales as their MPEC counterparts and represented a highly relevant comparator group for the MPEC genomes. (Strain information is given in Table S1 in the supplemental material.) By estimating the pan-genome content of this group of bacteria, we can detect genes that are present in the core genome of MPEC (defined as present in at least *n* − 1 of the sample) and yet are not present in the core genome of DFEC (defined as present in less than *n* − 1 of the sample). In doing so, this analysis can reveal factors that are ubiquitous in—and potentially necessary for—MPEC and yet are not strictly necessary for the survival of E. coli in the dairy farm environment. This analysis yielded the identification of just 45 genes which met these criteria (Fig. 1). Subsequently, by employing Fisher’s exact test to determine which of these genes were significantly more likely to be found in MPEC than in DFEC, we determined that just 8 genes achieved statistical significance (adjusted *P* < 0.05). These genes included the 7 coding sequences for the *fec* locus (*fecIRABCDE*) along with an adjacent gene, *yghV*, which forms part of the KpLE2 phage-like element which can mobilize the *fec* genes, even between different species of bacteria (12). The locus *fecIRABCDE* was absent in only one MPEC strain in our panel.

10.1128/mBio.00423-18.4 TABLE S1  Strain information and sequence accession numbers. Download TABLE S1, XLSX file, 0.01 MB. Copyright © 2018 Blum et al. 2018 Blum et al. This content is distributed under the terms of the Creative Commons Attribution 4.0 International license.

10.1128/mBio.00423-18.1 FIG S1  The population structure of E. coli. The figure shows phylogenetic relationships among 4,022 sequenced E. coli genomes based on the concatenated sequences of 201 common genes and estimated via maximum likelihood using RAxML. Download FIG S1, DOCX file, 0.04 MB. Copyright © 2018 Blum et al. 2018 Blum et al. This content is distributed under the terms of the Creative Commons Attribution 4.0 International license.

### Expression of the Fec system is induced in milk *in vitro* and *in vivo.*

**FIG 2:**
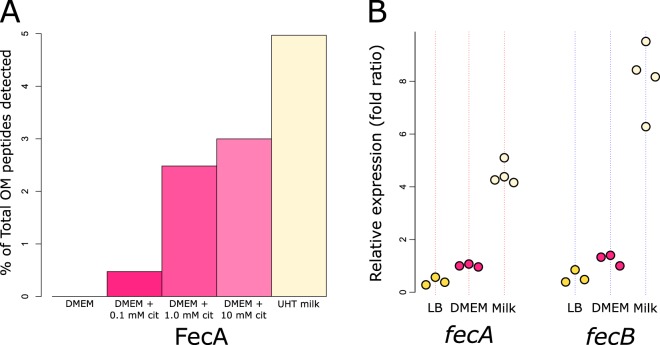
Fec system products and genes are upregulated in milk *in vitro* and *in vivo*. (A) Outer membrane (OM) protein preparations from MPEC strain P4 following growth in DMEM with or without citrate (cit) or UHT milk, showing citrate-dependent expression of FecA in the OM, with maximum expression achieved in milk. This experiment was performed once. (B) Transcription of *fecA* or *fecB* measured by RT-qPCR following growth in laboratory media (LB and DMEM) or following growth *in vivo* in the udders of experimentally infected cows relative to transcription of the control 16S and *frr* genes. Points represent biological replicates.

The strong correlation seen with regard to the presence of *fecIRABCDE* in MPEC genomes prompted us to investigate the expression of the Fec system during the infection process. The ability of E. coli to grow in milk is highly correlated with the ability to cause mastitis (13, 14), and so to explore this hypothesis, we employed a proteomic approach to validate the production of FecA during growth in milk *in vitro* and a transcriptional approach to investigate the expression of *fecA* and *fecB in vivo*. In our *in vitro* experiment, we grew prototypical MPEC strain O32:H37 P4 in Dulbecco’s modified Eagle’s medium (DMEM) with and without the addition of citrate, and in ultra-heat-treated (UHT) milk, and investigated the proteins present in the outer membrane (OM) of the bacteria by mass spectrometry. These data show that the OM protein FecA could not be identified following growth in DMEM without the addition of citrate (0 peptides). With the addition of 0.1 mM citrate, we could identify 10 peptides covering 20.3% of the FecA protein sequence. With the addition of 10 mM citrate, we could identify 50 peptides, covering 67.2% of FecA, and following growth in milk we could identify 63 peptides, covering 76% of the FecA protein sequence. These data are presented in Fig. 2A as a function of the total number of peptides identified in the OM preparations. Remarkably, in UHT milk, peptides from FecA constituted almost 5% of the total number of identified OM peptides. The use of 10 mM citrate in this experiment reflects the concentration of citrate found in milk (7). The list of all proteins identified is presented in Table S2. In our *in vivo* experiment, we experimentally infected cows with MPEC strain P4 and measured the transcript level of the outer membrane receptor gene *fecA* and the inner membrane transport component gene *fecB* by reverse transcription-quantitative PCR (RT-qPCR) in bacteria recovered from the milk at 6 h postinfection. We compared the levels of transcription of these genes to those observed in standard laboratory medium (DMEM or LB) (Fig. 2B). The data showed a clear upregulation of the *fecA* and *fecB* genes in the milk of experimentally infected cows relative to the results seen with standard laboratory media. Together, these data provide strong evidence that FecA is expressed and, given that our proteomic data were derived from an OM preparation, that it is localized to the outer membrane during growth in milk *in vitro* and that MPEC strains employ the Fec system *in vivo* in cows during the infection process.

10.1128/mBio.00423-18.5 TABLE S2  List of all outer membrane proteins identified by mass spectrometry. Download TABLE S2, CSV file, 0.2 MB. Copyright © 2018 Blum et al. 2018 Blum et al. This content is distributed under the terms of the Creative Commons Attribution 4.0 International license.

### The Fec system is essential for growth in milk and for experimental mastitis.

Considering the clear upregulation of Fec system genes and products induced by milk *in vitro* and *in vivo*, we requeried our previously published data on the ability of wild-type (WT) E. coli to grow in milk (13) with additional WT DFEC and MPEC isolates and discovered a good correlation between carriage of the *fecIRABCDE* locus and the ability of E. coli to support high levels of growth in milk (see Fig. S2 in the supplemental material). These data represent compelling circumstantial evidence that the Fec system is a key driver of the MPEC phenotype. To investigate further, we took two complementary approaches. First, we deleted the *fecIRABCDE* genes in MPEC strain P4 (P4 Δ*fecIRABCDE*), which has previously been well documented to grow well in milk and elicit mastitis (14). Second, we introduced the *fecIRABCDE* locus in *trans* into environmental isolate K71 (K71+*fecIRABCDE*), a strain which has been previously shown to be unable to grow to high levels in milk and to be incapable of eliciting experimental mastitis in mice (14) and cows (15).

10.1128/mBio.00423-18.2 FIG S2  The growth of wild-type strains in milk. Growth rates of wild-type strains in milk were measured by colony counting at 0, 4, and 8 h postinoculum. Strains encoding the *fecIRABCDE* genes are colored red and connected with red lines. Strains lacking the *fecIRABCDE* locus are colored blue and connected by blue lines. Strains isolated from mastitis (MPEC) are represented as squares (all *fec*^+^), and strains isolated from the dairy farm environment (DFEC) are represented as circles. These data reveal that the presence of the *fecIRABCDE* genes is highly correlated with the ability of E. coli to grow well in milk. Download FIG S2, DOCX file, 0.1 MB. Copyright © 2018 Blum et al. 2018 Blum et al. This content is distributed under the terms of the Creative Commons Attribution 4.0 International license.

**FIG 3:**
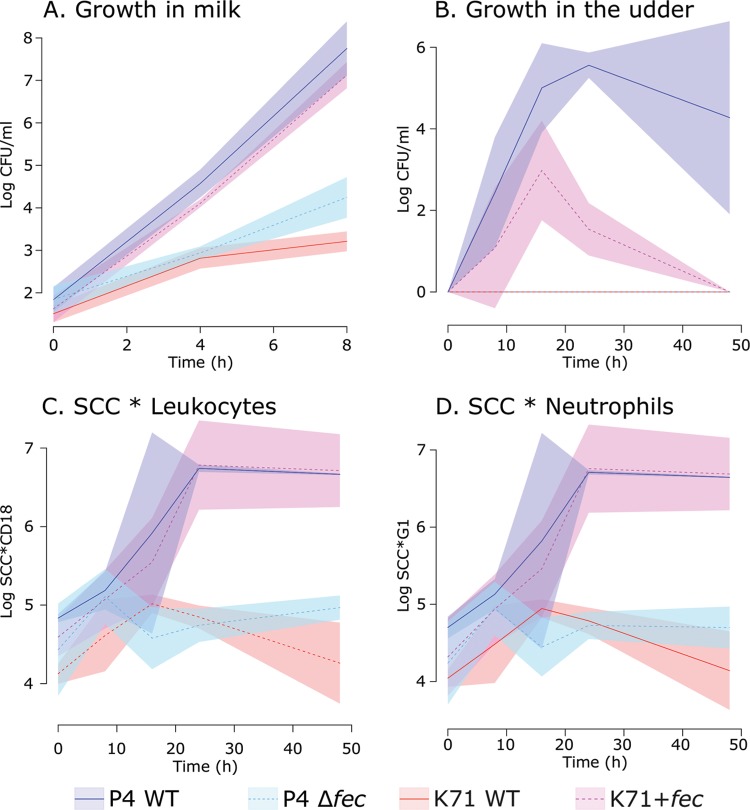
E. coli bacteria are dependent on *fecIRABCDE* for growth in milk and to elicit mastitis in cattle. (A) Growth in milk *in vitro* of *fecIRABCDE-*positive strains, the wild-type P4 strain (P4 WT), the K71 strain with introduction of *fecIRABCDE* (K71+*fec* mutant), and *fecIRABCDE-*negative strains (P4 Δ*fec* mutant and K71 WT). Introduction of the *fec* locus in K71 alone allowed growth in milk similar to that seen with MPEC P4. Deletion of the *fec* locus in P4 impaired growth in milk in a manner similar to that seen with the K71 WT strain naturally lacking *fec*. (B) Recovery of E. coli in milk of experimentally infected cows, showing an absolute requirement for *fecIRABCDE* to permit growth in the mammary gland *in vivo*. Strains lacking the *fec* locus (P4 Δ*fec* and K71 WT) were not detectable at any time point following intramammary infusion of bacteria (time zero), in contrast to the *fecIRABCDE*-positive strains (P4 WT and K71+*fec*). (C and D) Measurement of leukocytes (C) and neutrophils (D) as a function of total somatic cell counts (SCC) in the milk of challenged animals, showing that infection with *fecIRABCDE*-positive strains (P4 WT or K71+*fec*) led to a considerable influx of immune cells into the milk, coincident with symptoms of clinical mastitis. In the absence of *fecIRABCDE* (P4 Δ*fec* or K71 WT), levels of infiltration of immune cells into the milk were significantly lower, and no symptoms of mastitis were observed. Solid lines represent mean values, while the surrounding colored polygons represent standard deviations.

We investigated the ability of these strains to grow in milk. Figure 3A shows that in the absence of the Fec system, P4 suffered a four-log reduction in the number of cells generated over 8 h of growth. The level of growth of the wild-type K71 strain in milk was comparable to that seen with the P4 Δ*fecIRABCDE* strain. In contrast, the K71+*fecIRABCDE* strain showed an approximately five-log increase in biomass (measured by CFU) following 8 h of growth in milk and grew to levels comparable to those seen with the P4 wild-type strain. These data show that the presence of the *fecIRABCDE* genes is pivotal in allowing E. coli to grow in milk. The levels of growth of these strains were highly comparable following culture in standard laboratory media (Fig. S3).

10.1128/mBio.00423-18.3 FIG S3  The growth of wild-type P4 and K71, the P4Δ*fec* mutant, and K71 transformed with *fec* in standard laboratory media. These strains were cultured in nutrient broth for 20 h and measurements of optical density recorded. These data reveal that there was no difference in the growth rates of these strains in standard laboratory media. Lines represent the mean values for triplicate samples, and colored polygons represent standard deviations. Download FIG S3, DOCX file, 0.2 MB. Copyright © 2018 Blum et al. 2018 Blum et al. This content is distributed under the terms of the Creative Commons Attribution 4.0 International license.

Following from this, we used these strains in experimental infections in cows to investigate if dependence on *fecIRABCDE* for growth in milk translated to an effect on the incidence of mastitis *in vivo*. Over a time course, we collected milk from the udders of infected cows and profiled the number of viable E. coli bacteria by CFU (Fig. 3B) and the number of intramammary leukocytes (Fig. 3C) or neutrophils (Fig. 3D) by flow cytometry. Our results indicate that *fecIRABCDE* provides a major advantage with respect to the ability of E. coli to colonize and grow in the mammary gland and that the presence of that locus appears to be an absolute requirement. In the absence of *fecIRABCDE*, the P4 Δ*fecIRABCDE* strain and the wild-type K71 strain were not detectable by CFU analysis at any time point during our investigation. In contrast, both the wild-type P4 and K71+*fecIRABCDE* strains grew to substantial levels in the udders of cows during our experimental infection. There was an observable difference between these two *fecIRABCDE*-positive strains in the levels of growth, with the K71+*fecIRABCDE* strain not achieving the same biomass as wild-type P4 and appearing to be cleared more rapidly. For this reason, the stability of the Fec plasmid construct in transformed K71 was tested in batch culture in milk over a period of 36 h, in order to span the time period when a decrease of K71 Fec^+^ mutants was observed *in vivo*. No significant difference in the numbers of chloramphenicol-resistant (K71 Fec^+^ transformants) or total E. coli cells was observed *in vitro*. Thus, the plasmid appears to have remained stable in the overall population of K71 transformants and other factors may have played a role in bacterial clearance. Other genomic and phenotypic distinctions between E. coli strains K71 and P4 which may have contributed remain speculative and subject to separate systematic investigation.

Despite the difference in growth levels, there were broad similarities in the numbers of immune cells such as leukocytes (Fig. 3C) and neutrophils (Fig. 3D) infiltrating the milk following infection with either the wild-type P4 strain or the K71+*fecIRABCDE* strain, indicating that an inflammatory process was evoked by both strains, as such increases in immune cell infiltration into milk are typical of mastitis, although variable (16). In contrast, both of the *fecIRABCDE*-negative strains (P4 Δ*fecIRABCDE* and wild-type K71) were defined by the presence of a considerably lower level of immune cell infiltration than was seen with their *fecIRABCDE*-positive counterparts, suggesting that neither strain elicited an inflammatory response in the challenged mammary glands. These infection studies showed that Fec substantially enables fitness, promoting growth and allowing intramammary infection and, consequently, mastitis.

## DISCUSSION

In this paper, we present for the first time powerful evidence for the significant contribution of the Fec iron-citrate uptake system as a highly significant genetic determinant for the ability of E. coli to successfully cause intramammary infection and thus to elicit mastitis. Here, we show that the Fec system is a prerequisite for the pathogenicity of E. coli in the mammary gland, as the prototypical MPEC strain P4 is incapable of eliciting mastitis in its absence and the demonstrably non-mammary gland-pathogenic K71 strain gains MPEC capability with its addition. Although the *fec*-positive strains grew at comparable levels in milk *in vitro*, there were clear differences in the level of biomass achieved by the K71+*fecIRABCDE* strain relative to that achieved by the wild-type P4 strain *in vivo*. This lower growth capacity *in vivo*, which is unlikely to have resulted from the loss of the plasmid carrying *fecIRABCDE* (for which we did not find any evidence), may be presumed to improve efficiency of bacterial cell washout through milking, which could exacerbate fitness deficiencies in the K71 transformant population. Nonetheless, the bovine udder is an environment that, in the absence of *fecIRABCDE*, strain K71 has not previously been found to be able to exploit. This locus provides functional capability that retrieves this strain from an inherent fitness deficit in intramammary colonization and growth. Therefore, we consider it is likely that MPEC strains, exemplified by P4, are highly adapted for selection, colonization, and growth in the bovine udder, a capability to which Fec makes a substantive contribution.

Previous studies by us and others showed that E. coli consistently carried the high-affinity enterobactin iron acquisition system, and this is the case with both P4 and K71 strains. In fact, K71 also possesses a locus encoding aerobactin (frequently associated with extraintestinal E. coli but not with mastitis strains); hence, an inability to acquire iron by systems other than Fec cannot predicate the differences between P4 and K71 strains. We postulate that the presence of selective pressure with respect to the ability of P4 to successfully compete in bovine milk has resulted in a strain which is considerably more fit in this niche than the K71+*fecIRABCDE* strain. For instance, K71 was shown to be less resistant than typical MPEC to phagocytosis and killing by leukocytes *in vitro*, which could have contributed to faster clearance of this strain in the mammary gland, after the onset of mastitis (which is accompanied by significant levels of inflammatory cell influx) (13). Overall, our data indicate that the *fecIRABCDE* genes provide E. coli with a necessary “foot in the door” to bovine mastitis, allowing the possibility that the bacteria would establish in and adapt to this niche, rather than being excluded *a priori*.

The inflammatory reaction in the mammary gland is triggered by microbe-associated molecular patterns (MAMPS) of E. coli. The best characterized is that seen with lipopolysaccharide (LPS) (endotoxin), which, when infused alone in the mammary gland, causes an inflammatory reaction resembling actual E. coli infection although shorter in duration. Other E. coli MAMPs were shown to also play a role in triggering inflammation in the gland (17), although LPS is the most highly potent. Factors that improve E. coli fitness in the gland milieu, allowing some strains to be better adapted to this niche, appear to affect pathogenicity. For example, some strains have been shown to be able to invade mammary epithelial cells, with this ability being associated with E. coli causing persistent infections (18).

The genome of strain K71 harbors various virulence-associated factors, such as the type VI secretion system, type IV pilus, fimbriae, and curli adhesins, some of which have also been described in MPEC (14, 19, 20). Also, although lacking the Fec cluster, strain K71 has in common with MPEC (including P4) the iron uptake systems enterobactin and *efeUOB* and also has aerobactin, which is uncommon among MPEC strains but common among extraintestinal pathogenic E. coli (ExPEC) strains (20). Thus, in terms of virulence-associated gene content, strain K71 has other determinants associated with potential pathogenicity and yet cannot elicit mastitis in the absence of the Fec locus. Therefore, it is expected that presumptive virulence factors of E. coli K71 play roles in colonizing the bovine udder, albeit with the Fec system playing a preemptive role.

We conclude that the Fec system is essential to confer to E. coli the ability to cause mastitis. We propose that, without Fec, E. coli cannot multiply in the bovine milk in the mammary gland sufficiently to allow establishment of an actual infection and consequent triggering of inflammation. E. coli mastitis is often considered to be “environmental” in origin, and this is supported by our observation that some DFEC strains carry *fecIRABCDE* and can grow in milk to levels comparable to those seen with MPEC. We consider it likely that MPEC strains are selected from *fecIRABCDE-*carrying E. coli present in the environment. It is debatable whether the Fec system should be considered a virulence factor or a fitness factor in this context and, consequently, whether it defines MPEC as an E. coli pathotype or as an ecotype. In contrast to the intestinal milieu, where a variety of nonpathogenic strains may exist, the mammary gland is normally devoid of any E. coli bacteria, and there are no “mammary commensal” strains. In this niche, E. coli bacteria possessing the Fec locus have a fitness advantage which facilitates adaptation and growth in this milieu. Together with this capability, there are further fitness and virulence determinants which contribute to characteristics such as resilience with respect to host antimicrobial defenses and, of course, the major contribution from endotoxin as bacterial numbers rise. These characteristics show similarities with phylogenetically distinct extraintestinal pathogenic E. coli (ExPEC) strains such as uropathogenic E. coli (UPEC). Like MPEC, to date, no single essential virulence factor has been defined in ExPEC but, rather, a suite of systems (some more important than others), all enabling colonization of the urinary tract or other extraintestinal sites, has been described (8). Also similarly to MPEC, many E. coli also harbor those same systems, and yet it is the ability of ExPEC to utilize them outside the gut, and particularly in the urinary tract or in blood circulation, that allows pathogenicity in this specific site. This supports the definition of MPEC as a distinct clade among ExPEC.

Our data represent a validation of the postgenomics method and set an example for how comprehensive population-level genomic analyses can reveal crucial factors which underpin the ability of bacteria to cause complex infections. Further study is needed to investigate how this new information on the role of the Fec system in MPEC can be explored to provide novel effective management strategies or efficacious treatments for mastitis. Interestingly, Klebsiella pneumoniae bacteria, specifically, those isolates collected from cows with mastitis, also carry the *fecIRABCDE* locus (10, 21), suggesting that our results reported here on the importance of citrate and ferric citrate sensing mechanisms may have extended relevance for other mastitis-causing organisms.

## MATERIALS AND METHODS

### Ethics statement.

The animal experiments received approval from the Ethics Committee of Val de Loire, France (DGRI’s APAFIS agreement no. 813-2015061109103810v2), and were compliant with all applicable provisions established by European directive 2010/2063/UE (bovine intramammary challenge for RT-qPCR) or were approved by the ARO Committee of Animal Experimentation and adhered to stipulated ethical and safety guidelines (bovine intramammary challenge for pathogenicity assay).

### Isolation of E. coli strains and genome sequencing.

E. coli isolates were collected and identified as described before (13, 22). MPEC (mammary pathogenic E. coli) isolates were collected from mammary glands of cows with clinical mastitis. MPEC samples were selected only from cultures with clear E. coli growth, without contaminants, and at least two consecutive cultures were performed to confirm E. coli etiology in mastitis cases. DFEC (dairy farm E. coli) strains were isolated from different places in the cowshed in the same farms and during the same periods of time as the MPEC strains.

Bacteria were grown in blood agar overnight. Total DNA was extracted from each of the isolates using a MasterPure DNA purification kit (Epicentre, Madison, WI) per the manufacturer’s instructions. Sequencing was performed using an Illumina MiSeq sequencer at Glasgow Polyomics, Wolfson Wohl Cancer Research Center, Glasgow, United Kingdom, as previously described (11).

### E. coli pan-genome estimation.

The pan-genome structure of the E. coli genome sequences was estimated as described recently (11). Briefly, the genes for the 62 E. coli isolates used in this study were predicted by Prodigal (23). A preclustering step was carried out, via iterative NUCmer alignments of successive genomes to the pan-genome, to assemble a bank of genes that shared less than 95% sequence similarity (an average of percent sequence identity and percent matching length). A clustering step was then carried out (via NUCmer) to identify gene families which shared less than 80% sequence similarity. These data were transposed to a presence/absence matrix for further investigation. We extracted genes that were part of the core MPEC genome (defined as present in at least *n* − 1 genomes) and yet were not part of the DFEC core genome (defined as absent in at least *n* − 1 genomes) and investigated the difference in carriage between MPEC and DFEC by Fisher’s exact test. We corrected the resulting *P* values for multiple tests using the Hochberg method implemented using the “p.adjust” function within R.

### E. coli phylogenetic analysis.

The structure of the pan-genome was explored to identify genes present in all strains. This resulted in the identification of 3,218 genes. The nucleotide sequences for these genes were aligned by Muscle (24) and concatenated, and then poorly aligned regions were eliminated using Gblocks (25). The resulting 2.8-Mbp alignment per sequence was used to build a maximum likelihood tree using RAxML (26) under the GTR model with 1,000 bootstrap replicates. Our population analysis of 4,022 E. coli genomes was carried out as described in a recent publication (27).

### Generation of *fecIRABCDE* gene deletions in E. coli P4*.*

A nonpolar gene deletion of the *fec* locus was generated using the Lambda Red-mediated recombination method (28). Briefly, template plasmid pKD4 (conferring kanamycin resistance [kanamycin^r^]) was used to amplify the corresponding resistance cassette with primers containing flanks directly adjacent to the *fec* locus (Table S3). PCR products were purified by phenol-chloroform extraction, ethanol precipitation, and DpnI treatment to concentrate the template and maximize the efficiency of the recombination reaction. E. coli P4 carrying pKD46 was cultured to an optical density (OD) of 0.4 in super optimal broth (SOB) at 30°C and induced with 10 mM arabinose 1 h prior to being made electrocompetent by washing and 1:100-fold dilution in ice-cold 10% glycerol. Cells were then transformed with 1 μg of template per 50 μl and recovered for 2 h at 37°C in super optimal broth with catabolite repression (SOC) before plating on selective media was performed. Recombinants were selected for on LB agar containing 40 μg/ml kanamycin. Phenotypically positive colonies were screened for resistance cassette recombination by PCR. Removal of template resistance cassettes was performed by the use of a temperature selection shift from 30°C to 37°C on plain LB media using plasmid pCP20 (ampicillin^r^). Removal of the *fecIRABCDE* locus was confirmed by PCR.

10.1128/mBio.00423-18.6 TABLE S3  *fec* locus mutagenesis primers for E. coli P4 (underlined sequence corresponds to the pKD4 template plasmid sequence). Download TABLE S3, DOCX file, 0.01 MB. Copyright © 2018 Blum et al. 2018 Blum et al. This content is distributed under the terms of the Creative Commons Attribution 4.0 International license.

### Construction of the *fecIRABCDE* plasmid and K71 transformation.

Complementation of the *fec* locus mutant was achieved by expression of *fecIRABCDE* from its natural promoter on a modified pACYC184 backbone. The complementation plasmid was constructed using the NEBuilder protocol (New England Biolabs). Briefly, pACYC184 was linearized by PCR to create a blunt end backbone (2,755 bp) lacking the tetracycline^r^ gene. Two fragments corresponding to the two halves of the *fecIRABCDE* locus plus an additional 200 bp from either side (4,003 bp and 3,867 bp, respectively) were also amplified by PCR using primers that created unique orientation-specific overhangs for assembly of both halves on the backbone of pACYC184. Primers were designed using the NEBuilder design module, and the design strategy for assembly was confirmed using MacVector version 12.5. PCR fragments were purified by gel extraction and used in a one-step assembly reaction according to the NEBuilder kit manufacturer’s protocol. Reaction mixtures were transformed and selected on LB agar containing 20 μg/ml chloramphenicol. Positive clones were confirmed by PCR and digestion with EcoRV. The construct contained a unique EcoRV restriction site, yielding a 10,625-bp linearized fragment. A table of the primer sequences used is presented in Table S4.

10.1128/mBio.00423-18.7 TABLE S4  Primer sequences used to generate *fecIRABCDE* complementation plasmid. Download TABLE S4, DOCX file, 0.01 MB. Copyright © 2018 Blum et al. 2018 Blum et al. This content is distributed under the terms of the Creative Commons Attribution 4.0 International license.

K71 was transformed with *fecIRABCDE* plasmid construct by electroporation. Chloramphenicol-resistant clones were compared to wild-type K71 by enterobacterial repetitive intergenic consensus-PCR (ERIC-PCR) (29) and plasmid profiling (30) to confirm their identification.

### Proteomics.

E. coli strain P4 was grown in DMEM (Sigma) overnight and inoculated at an OD_600_ of 0.001 and then grown for 6 h aerobically at 37°C under static conditions in (i) DMEM; (ii) DMEM supplemented with 0.1, 1.0, or 10 mM citric acid; or (iii) UHT milk. DMEM cultures were supplemented with 100 mM HEPES (pH 7.2). Outer membrane proteins were prepared via Sarkosyl extraction and analyzed via shotgun proteomics as previously reported (31).

### Milk growth experiments.

The rate of growth in milk was tested as described before (14). Briefly, bacteria were harvested from overnight growth on blood agar, or on LB agar supplemented with chloramphenicol for strain K71+*fecIRABCDE*, and washed with phosphate-buffered saline (PBS). The bacterial concentration was assessed by calculation of the level of turbidity against a previously prepared concentration × the OD curve and was normalized to approximately 2 × 10^3^ CFU/ml. Fresh bovine milk was collected 1 day prior to the experiment, pasteurized at 80°C for 10 min, and tested for sterility by culture on blood agar. A 100-μl volume of bacterial suspension was inoculated in 40 ml of milk, and the mixture was incubated at 37°C. Samples were aseptically collected for CPU determinations at 4 and 8 h. Experiments were conducted up to four times separately, with two replicates per experiment. The numbers of data points per time point were 8 (P4 WT strain), 6 (strain P4 Δ*fec* and strain K71 WT), and 4 (strain K71+*fec*). The stability of the Fec plasmid construct in transformed strain K71 was tested *in vitro* in a batch milk culture of this mutant grown up to 36 h. In this experiment, to check for any growth of K71 that was independent of the plasmid, bacterial counting was performed on agar with and without chloramphenicol. P4 WT and K71 WT were used as positive (high-growth) and negative (lower-growth) controls.

### Bovine intramammary challenge for RT-qPCR.

Four Holstein-Frisian cows were challenged as previously described (32). At 8 h postinoculation, 50 ml collected milk was mixed with 100 ml RNAProtect (Qiagen). The suspension was incubated at room temperature for 5 min and then centrifuged for 20 min at 5,000 × *g*. The supernatant was discarded and the pellet stored at −20°C. Prior to RNA extraction, the pellet was thawed on ice and resuspended in 1 ml of Trizol reagent. The suspension was transferred to a 2-ml Lysing Matrix B tube (MP Biomedicals) and shaken for 45 s at speed 6 in a FastPrep 24 apparatus (MP Biomedicals). A 200-μl volume of chloroform was added to each tube. After 5 min at room temperature, tubes were centrifuged for 15 min at 12,000 × *g* at 4°C. The aqueous phase was collected, mixed with an equal volume of 70% ethanol, and loaded onto a NucleoSpin RNA column (Macherey-Nagel). RNA was then extracted according to the manufacturer’s instructions with on-column DNase 1 treatment. RNA extraction from *in vitro* cultures was performed using E. coli P4 grown overnight in a static culture at 37°C in DMEM or LB broth.

Approximately 100 ng of RNA was converted to cDNA using iScript Reverse Transcription Supermix (Bio-Rad). Quantitative PCR was then performed using iQ SYBR green Supermix (Bio-Rad) and primers for *fecA* (F, AACGCCAACGCTTGATAACG; R, AGCGTGCCTTTATGTTTCGG) or *fecB* (F, ACATCGCCTTGCAGCAAATC; R, ACCATTTCGCCGATGATAGC). Quantification was performed using expression levels of the 16S gene (F, GTTAATACCTTTGCTCATTGA; R, ACCAGGGTATCTAATCCTGTT) and *frr* (F, TGGACAAATGCGTAGAAGCG; R, ATTCCACGACAATGCCATCC) as the reference genes, alongside the Genex macro (Bio-Rad).

### Bovine intramammary challenge for pathogenicity assay.

Intramammary challenge was performed in Holstein cows. All mammary glands used were free of infection based on three consecutive negative bacteriologic examinations and a milk somatic cell count (SCC) level of lower than 100,000 cells/ml. Inocula were prepared by harvesting bacteria from a culture grown overnight on blood agar (P4Δ*fecIRABCDE*) or on LB agar containing chloramphenicol (K71+*fecIRABCDE*). Bacteria were washed in pyrogen-free saline (PFS), and quantification of viable bacteria was estimated by optical density and confirmed by plate counting. The inoculum was stored at 4°C for 10 h. The bacterial cell number was adjusted using PFS just before challenge to yield an inoculum of approximately 100 CFU/ml and was confirmed by plate counting. Before morning milking (time zero), a milk sample was taken from each gland. After milking, teats were cleaned and disinfected with iodine and antiseptic cloths, and the inoculum was injected through the teat canal using sterile syringe mounts for intramammary injection. Cows were observed, including rectal temperature measurements, for 48 h for clinical signs of mastitis. Strains K71+*fecIRABCDE* and P4Δ*fecIRABCDE* were inoculated into glands from seven cows in total. Strains were inoculated into contralateral glands in four animals, whereas strain K71+*fecIRABCDE* was inoculated into a single gland in one animal and strain P4Δ*fecIRABCDE* was inoculated into a single gland in two animals. Due to the constraints imposed by the high cost of animal challenges, control data for strains P4 and wild-type K71 were taken from previous work (15). We state explicitly that these strains were not used concurrently with their respective mutants; however, the experiments were conducted identically.

Samples of milk from challenged glands were collected before milking and after 8, 16, 24, and 48 h. Teats were cleaned and disinfected, and foremilk was discarded. A sample of 3 ml of milk was collected aseptically into sterile tubes for bacteriologic culture. To detect contaminants, bacteriologic culture was performed by plating 100 µl of milk on blood agar. E. coli counting was performed on MacConkey agar by CFU counting, following serial dilutions of milk with saline solution. Approximately 200 ml of milk was then collected for SCC determinations, and differentiation of leukocytes was evaluated as described previously (3).

### Accession number(s).

Genome sequences were uploaded to NCBI under the accession numbers given in Table S1. The NCBI BioProject accession number for this study is PRJNA341825.
