# Supplementary material for: Postgenomics Characterization of an Essential Genetic Determinant of Mammary Pathogenic Escherichia coli
Source: mBio. 2018 Apr 3;9(2):e00423-18. doi: 10.1128/mBio.00423-18 (PMC5885034; doi:10.1128/mBio.00423-18)
Supplement: FIG S1 [file mbo002183806sf1.docx]

**Figure S1. The population structure of *E. coli*.** This tree represents the phylogenetic relationships between 4022 sequenced *E. coli* genomes, estimated via maximum likelihood using RaxML. This tree is based on the concatenated sequence of 201 common genes. The position of strain DFEC K71 is indicated by a blue asterisk, and MPEC P4 by a red asterisk.
