# Supplementary material for: Postgenomics Characterization of an Essential Genetic Determinant of Mammary Pathogenic Escherichia coli
Source: mBio. 2018 Apr 3;9(2):e00423-18. doi: 10.1128/mBio.00423-18 (PMC5885034; doi:10.1128/mBio.00423-18)
Supplement: FIG S2 [file mbo002183806sf2.docx]

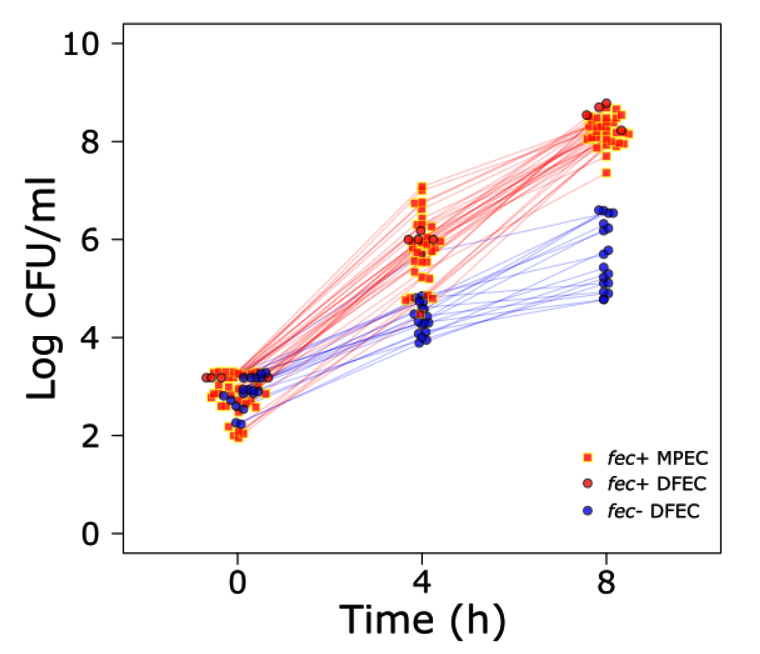


**Figure S2. The growth of wild-type strains in milk.** In this figure, wild-type strains have been cultured in milk for up to 8 hours, with samples taken for colony counting at 0, 4, and 8 hours’ post inoculum to measure growth rate. Strains encoding the *fecIRABCDE* genes are coloured red and connected with red lines. Strains lacking the *fecIRABCDE* genes are coloured blue and connected by blue lines. Strains isolated from mastitis (MPEC) are represented as squares, and strains isolated from the dairy farm environment (DFEC) are represented as circles. These data reveal that the presence of the *fecIRABCDE* genes can be highly correlated with the ability for *E. coli* to grow well in milk.
