# Supplementary material for: Postgenomics Characterization of an Essential Genetic Determinant of Mammary Pathogenic Escherichia coli
Source: mBio. 2018 Apr 3;9(2):e00423-18. doi: 10.1128/mBio.00423-18 (PMC5885034; doi:10.1128/mBio.00423-18)
Supplement: FIG S3 [file mbo002183806sf3.docx]

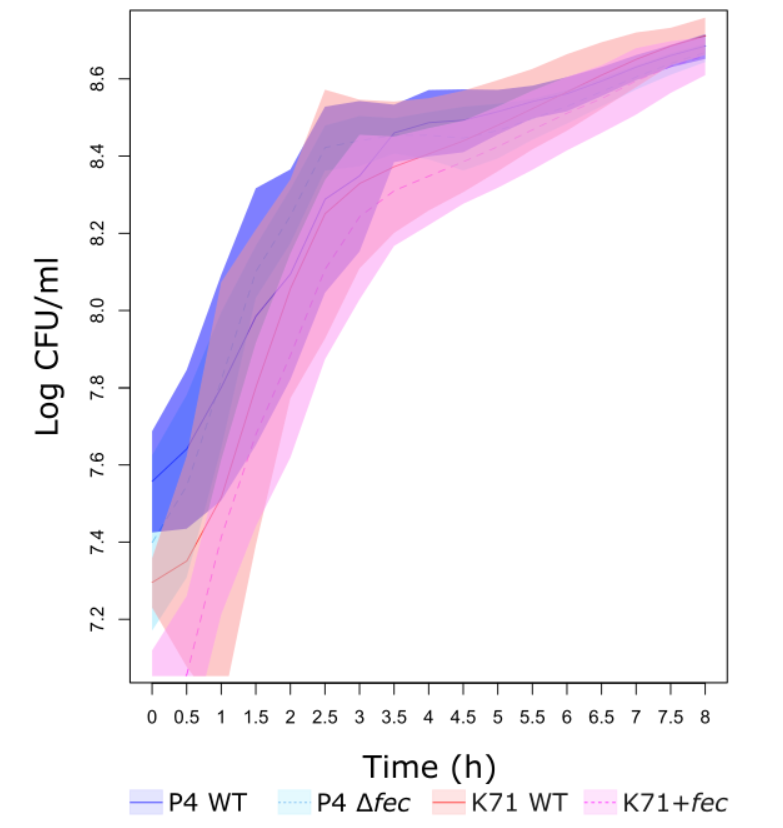


**Figure S3a. The growth of wild type P4 and K71, the P4 *fec* mutant and K71 transformed with *fec* in standard laboratory media.** This data reflects an approximation of the Log CFU/ml growth of *E. coli* strains over 8 hours in standard laboratory media to compare with figure 3 in the main text. Lines represent the mean value of triplicate readings and polygons represent standard deviation. These data are extrapolated from the OD_600_ readings displayed in Figure S3b under the assumption that an OD_600 ­_of 1 equates to roughly 8*10^8 CFU/ml.


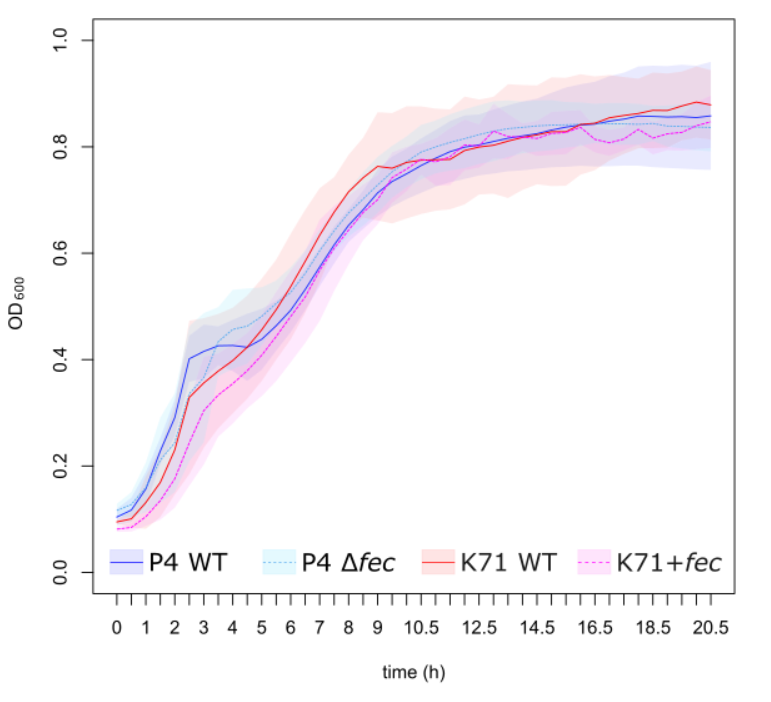


**Figure S3b. The growth of wild type P4 and K71, the P4 *fec* mutant and K71 transformed with *fec* in standard laboratory media.** These strains were cultured in nutrient broth for 20 hours and measurements of optical density recorded. These data reveal that in standard laboratory media there is no difference in the growth rate of these strains. Lines represent the mean value for triplicate samples, coloured polygons represent standard deviation.
