# Supplementary material for: Postgenomics Characterization of an Essential Genetic Determinant of Mammary Pathogenic Escherichia coli
Source: mBio. 2018 Apr 3;9(2):e00423-18. doi: 10.1128/mBio.00423-18 (PMC5885034; doi:10.1128/mBio.00423-18)
Supplement: TABLE S3 [file mbo002183806st3.docx]

| Name |  |
| --- | --- |
| **fecIRABCDE_mutagenesis_F** | ATTCTCATATTAATATGACTACGTGATAATTAACTTTTGATGCACTCCGCGTGTAGGCTGGAGCTGCTTC |
| **fecIRABCDE_mutagenesis_R** | GCCAGTCCAGTTTCATTCAGTCGTGGTTTGGTTCTTACGGCCTGTGCAATCATATGAATATCCTCCTTAG |
| **fecIRABCDE_check_F** | AAAAATGTCTATTGGAAACAA |
| **fecIRABCDE_check_R** | TGGTAATCTTTCAGAGTCCG |

Table S3. *fec* locus mutagenesis primers for *E. coli* P4 (underlined sequence corresponds to the pKD4 template plasmid sequence).
