# Supplementary material for: Postgenomics Characterization of an Essential Genetic Determinant of Mammary Pathogenic Escherichia coli
Source: mBio. 2018 Apr 3;9(2):e00423-18. doi: 10.1128/mBio.00423-18 (PMC5885034; doi:10.1128/mBio.00423-18)
Supplement: TABLE S4 [file mbo002183806st4.docx]

| **Name** | **Sequence** |
| --- | --- |
| **pACYC_fec_backbone_F** | GCAGAACATATCCATCGC |
| **pACYC_fec_backbone_R** | CCGCATTAAAGCTTATCG |
| **fec_Frag1_pACYC_F** | ATCGATAAGCTTTAATGCGGAGTCATTCTGACCGACAC |
| **fec_Frag1_pACYC_R** | GATGCCTTTGTTGTTGTCGTCATAAGAGC |
| **fec_Frag2_pACYC_F** | ACGACAACAACAAAGGCATCTATGCAGG |
| **fec_Frag2_pACYC_R** | ACGCGATGGATATGTTCTGCCCTGTCAGCCTAAAGCAAG |

Table S4. Primer sequences used to generate *fecIRABCDE* complementation plasmid
